# Assessment and Disruption of Ruminative Episodes to Enhance Mobile Cognitive Behavioral Therapy Just-in-Time Adaptive Interventions in Clinical Depression: Pilot Randomized Controlled Trial

**DOI:** 10.2196/37270

**Published:** 2023-01-05

**Authors:** Liyuan Wang, Lynn Miller

**Affiliations:** 1 Children's Hospital, Los Angeles Department of Adolescent and Young Adult Los Angeles, CA United States; 2 University of Southern California Annenberg School for Communication and Journalism Los Angeles, CA United States

**Keywords:** depressive rumination, mobile health, mHealth, just-in-time adaptive intervention, depression, mental health, mobile phone

## Abstract

**Background:**

A just-in-time adaptive intervention (JITAI) is “designed to address the dynamically changing needs of individuals via the provision of the type or amount of support needed, at the right time when needed.” If and how rumination-focused cognitive behavioral therapy (RFCBT), *the gold standard*, blocks emotional cascades underlying rumination is unclear. Furthermore, cognitive behavioral therapy has been successfully used as a mobile variant, but RFCBT has not been adapted for a mobile variant (mobile RFCBT [MRFCBT]) or for a JITAI variant.

**Objective:**

This study aimed to pilot-test a fully automated JITAI leveraging RFCBT and ways to identify and block cascading depressive rumination.

**Methods:**

Patients in therapy for clinical depression were recruited for a randomized controlled trial (RCT). After consenting to be part of the RCT, they were randomly assigned to either of the 2 mobile versions of the RFCBT conditions personalized to the individual’s rumination timing patterns (JITAI-MRFCBT) or a no-treatment control condition through a double-blind procedure. Although the initial design was to have a 3-armed trial with 2 JITAI conditions (a JITAI and a narrative JITAI condition), we later opted to collapse those 2 conditions into 1 JITAI condition because of the low number of participants. All participants were recruited and participated through their smartphones, receiving 5 SMS text message reminders on each of the 35 days to self-report their rumination-related symptoms (eg, rumination episodes and duration). In the JITAI-MRFCBT condition, they also received treatment materials. The first 7 days provided a rumination baseline, and the last 7 days provided a postintervention rumination value. In total, 42% (25/59) of volunteers were eligible and provided their phone numbers, 20% (5/25) of whom never replied to the SMS text message reminding them to start the RCT. A total of 90% (18/20) of volunteers completed it (ie, finishing, as prespecified, 80% of the questionnaires and training tasks) and, therefore, were included in the analysis.

**Results:**

Using independent 2-tailed *t* tests with bootstrapping, results showed that participants in the JITAI-MRFCBT condition, compared with those in the control condition, reported a greater reduction in counts of rumination episodes (mean −25.28, SD 14.50 vs mean 1.44, SD 4.12, *P*<.001) and greater reduced average time (minutes) spent in rumination (mean −21.53, SD 17.6 vs mean 1.47, SD 1.5; *P*=.04). Results also suggest that, compared with those in the control group, those in treatment reduced ruminative carryover from one episode to the next.

**Conclusions:**

The results suggest that JITAI-MRFCBT may reduce negative rumination by providing RFCBT just in time following rumination, thereby blocking the next rumination episode using the same trigger. This study supports a subsequent, full-scale JITAI and the importance of leveraging mobile smartphone technology with MRFCBT to curb depressive symptoms.

**Trial Registration:**

ClinicalTrials.gov NCT04554706; https://clinicaltrials.gov/ct2/show/NCT04554706

## Introduction

### Background

Major depressive disorder (MDD) [1], or clinical depression, is a mood disorder associated with prevalent sadness, hopelessness, and anhedonia and often manifests symptoms associated with cognitive impairment and social functioning disability. A challenge underlying MDD is dysregulation of the emotional cascades underlying depressive rumination. Rumination, or depressive rumination response, is a thinking style defined as “the tendency to passively and repetitively focus on the experience of negative moods, as well as their causes and consequences” [2]. When rumination is driven by cascades of negative emotions, one can become more depressed and use more depressive rumination to cope with their depression [3]. Specifically, depressive rumination and the underlying emotional cascades usually stem from problematic interpersonal communication [3,4]. Negative verbal and nonverbal communicative patterns of depressed individuals can adversely affect the nature of social interactions and strain social networks [5,6]. Social impairments such as inhibited communication with family members and friends and the use of negative self-focused verbalizations have long been associated with negative psychological states, including depression [7]. In interpersonal relationships, depression-related patterns of rumination usually precede a subsequent problematic interpersonal communication episode, exacerbating levels of depression [7].

Cognitive behavioral therapy (CBT) has long been one of the chief promising face-to-face interventions in this domain [8]. Patients in CBT interventions learn ways to monitor and improve their psychological symptoms through a series of tasks such as learning how to use diaries, emotion regulation techniques, and mindfulness tools [9]. CBT offers a wide range of strategies that help patients maintain, trigger, and mitigate their depressive symptoms [9,10]. A number of meta-analytical reviews have shown that CBT has strong efficacy in mitigating depression outcomes and recommend it as the *gold standard* frontline treatment for depression [10-13].

### Just-in-Time Adaptive Interventions Using Rumination-Focused CBT to Reduce Depression

A number of meta-analytical reviews have suggested that people’s everyday technologies (eg, smartphones and wearable devices) can provide not only scalable but efficacious mental health treatment [14-16]. Specifically, those reviews point to the efficacy of CBT delivered via mobile phone (mobile CBT [MCBT]) through apps. MCBTs have the capacity to integrate “gold standard treatment (CBT)” to bolster and maintain psychological resilience with scalable mobile alternatives [14].

However, MCBTs are not without their limitations. Although MCBT apps can provide some treatment information, none of those apps currently have the functionality to further diagnose and provide customized in-the-moment feedback based on a given client’s specific symptoms [5]. This is due at least in part to a failure to typically use a customized within-person approach affording intensive measurements (ie, ≥100 measures per variable per person), which can be leveraged to track a given participant’s progress within a given context and across different situational contexts [17,18]. Therefore, there is a need for rumination-focused MCBTs (or mobile rumination-focused CBTs [MRFCBTs]) that are more specifically designed to address rumination via a mobile device and effectively use a within-person approach for personalized feedback to changes in client state.

There are many reasons why an intensive within-subject MRFCBT using a just-in-time adaptive intervention (JITAI) approach—affording cues for personalized delivery of in-the-moment diagnosis and feedback—matters. First, a given client with depression does not respond with rumination across all contexts. Therefore, MCBTs without intensive within-subject assessment offered by JITAIs could be perceived as nonresponsive to the changing state of the client with depression, which could undermine treatment effectiveness [19-22]. Second, an intensive within-person design could allow for the collection of multimodal information using noninvasive sensors and active self-reports. These rich data can enable computational possibilities to predict and prevent risks when participants are the most vulnerable. For example, ecological momentary assessments (EMAs), which actively measure people’s in-the-moment emotional reactivity to different stressful events at various time points [23-25], have also been shown to be reliable predictors of subsequent depressive outcomes. Third, it is unclear how rumination in one context for an individual with depression carries over into other contexts, exacerbating those effects as rumination within subjects is not even assessed—let alone used for potential markers for personalized feedback or interventions. Thus, current MCBT apps function more like a pool of knowledge for active information seekers rather than professional health care providers from which one can seek personalized and responsive help. Fourth, MRFCBT—with rumination treatment in mind [26]—has not been successfully tested, to our knowledge, in mobile form, let alone with JITAI features.

Clearly, there is a strong need for current MRFCBT apps to integrate JITAI features (ie, algorithms), taking advantage of multi-time assessments (both active and passive) to detect vulnerabilities and provide feedback at the exact moment of need [27]. To advance this science, this project used EMA, where such moment-to-moment assessments in real time have been successfully applied in numerous health science domains [28]. That work suggests that JITAI designs could be translated into MCBTs, including an MRFCBT. JITAIs tested by researchers without “real-time” human clinicians—that have given participants feedback based on their self-reported problems—have been found to be effective [5]. Therefore, this project was designed to test the efficacy of an MRFCBT with JITAI features in reducing ruminative thoughts—a major symptom of depression. To date, this has not been assessed. Specifically, we hypothesized (hypothesis 1) that a JITAI-delivered MRFCBT intervention would be more effective than a no-treatment control group (assessing rumination but not providing treatment) in reducing rumination over time.

### The Link Between Rumination and Depression

JITAI design can target highly intrapersonal symptoms that are otherwise hard to track. In particular, one’s ruminative responses (ie, trait rumination) to their immediate social interactions can contribute to one’s ruminative patterns and depression outcomes [29-31]. People with strong ruminative tendencies may find themselves subconsciously revisiting and recurrently thinking about some unpleasant interpersonal events. For example, after experiencing an interpersonal disagreement, those who are high in ruminative response may find themselves more likely to engage in thoughts concerning “Why did this happen to me,” “What was that person thinking of me,” and “Why couldn’t I handle this better.” Such ruminative processes usually lead individuals to recurrently revisit negative experiences, exacerbating negative affective states without producing strategies to improve future communication patterns [32].

Theories of depression [30,31,33] from both the cognitive and interpersonal or stress perspectives [4,34] have found that rumination over unpleasant experiences is a major reason for the increase in interpersonal conflict and the lack of relationship satisfaction, which subsequently leads to elevated depressive outcomes. In various studies on depression, the aftermath of depressive rumination is usually documented from an intrapersonal perspective. For example, using interviews, Lyubomirsky et al [29] found that, when engaging in more ruminative thoughts, people are more likely to generate biased interpretations of unpleasant events [29], and this usually elicits negative autobiographical memories [29], which exacerbate and prolong depression [35-37]. Without intensive assessments of one’s daily thought patterns, it would be impossible to track this highly intrapersonal process in terms of when and how it occurs, not to mention providing responsive treatment feedback for those who are in great need. With JITAIs and formative research on baseline rumination per participant, the occurrence of a ruminative episode could more easily be targeted via intensive measurements at multiple intervals.

## Methods

### Intervention System

The intervention system was created for a pilot study that, if successful, might lead to a subsequent full-scale randomized controlled clinical trial (RCT) nationally (ClinicalTrials.gov identifier NCT04554706). As a pilot RCT that precedes a large-scale trial, this project has some differences in sample size and measurement described in the preregistered trial. To be specific, this pilot is a 35-day–long, small-scale RCT with 2 between-subject conditions: a JITAI-MRFCBT condition (with personalized timing for participants’ own rumination patterns) and a no-treatment control condition with data collection to assess participant rumination but with no treatment. As the goal of this project is to focus on a major symptom of depression, which is excessive negative rumination, we adapted materials from rumination-focused CBT (RFCBT; materials on RFCBT were obtained through subscription to Psychology Tools [38]. The efficacy of RFCBT has already been tested in previous research [26]. MRFCBT includes activities, training exercises, and diaries that are designed to reduce ruminative episodes in patients with clinical depression [26]. Therapists can take advantage of the diaries provided by patients to offer tailored behavioral training (eg, reframing emotions, attribution perceptions, goal setting, and problem-solving) for patients. The RFCBT program is ideal for adaptation to MRFCBT with JITAI features: (1) the diary and rating systems with which therapists deliver tailored training sessions could be conveniently designed as questionnaires for participants to self-rate and document their conditions (providing also a 1-week baseline for personalized delivery, an adaptive feedback system sending treatment messages as participants progress into treatment, and a 1-week postintervention assessment), (2) the JITAI system can have the capacity to deliver automatic training sessions tailored to the results of individual self-reports, and (3) participants can have access to training materials and messages at the exact time of their requests. Therefore, 3 training material sets (ie, problem-solving, conflict attribution, and emotion regulation) from RFCBT were adapted into this JITAI condition. The JITAI conditions asked participants to document their ruminative episodes for 7 days. After that, they received intervention materials and training prompts for 3 weeks (21 days). They were asked to document their ruminative episodes for another week after completing the 3-week training materials (to compare the baseline experience with the one immediately after the intervention). It is worth pointing out that, as originally planned for the full registered RCT, we designed 2 of the JITAI conditions, one using the original adapted MRFCBT materials directly and the other taking a narrative approach to delivery of the MRFCBT. However, as we could not secure enough participants for the 2 JITAI conditions and there were no apparent differences between them in the pattern of findings (n=4 in the JITAI-MRFCBT condition and n=5 in the JITAI narrative RFCBT condition), we combined the 2 conditions as the JITAI condition for this pilot study. The no-treatment control condition did not have any training materials from the MRFCBT. Participants in this condition were only asked to document ruminative episodes (5 times a day) for 35 days.

**Figure 1:**
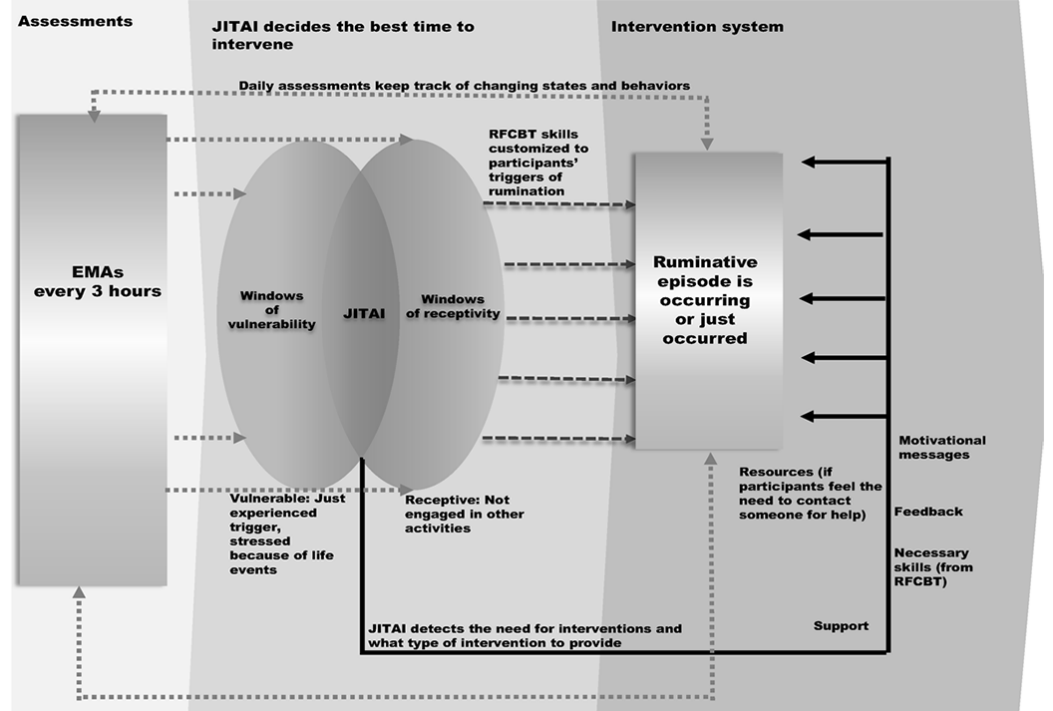
Just-in-time adaptive intervention (JITAI) design process.

Figure 1 is an illustration of the design of the JITAI system in this intervention. We used EMA as the main assessment tool to make decisions regarding which messages to deliver and when. EMA involved asking participants if they had experienced stressful events in the past 3 hours and, if so, what the events were (dotted double-headed arrows). If the participant identified the immediate presence of triggers of rumination episodes, the system prompted an inquiry regarding the nature of that trigger. At such trigger points, participants were quite vulnerable in terms of the probability of experiencing rumination episode events (dotted single-headed arrows). In addition, EMA measures, along with the previous baseline daily activity surveys, can determine whether participants are receptive to treatment at the moment of the rumination event (dotted single-headed arrows). If participants were not engaged in other activities (eg, driving and walking), they received JITAI feedback tailored to the type of trigger in the form of support, necessary problem-solving skills, motivation, and supportive messages (solid lines in Figure 1). In the meantime, we provided several sessions of RFCBT, adapted to (1) the screen size of a mobile phone and (2) readable length, when participants were receptive (dashed single-headed arrow).

Participants signed up and consented to take part before they were shown a link to the intervention website that asked participants to enter their phone numbers to start the RCT. This procedure was enabled by a programmable (through R; R Foundation for Statistical Computing) survey platform, Formr [39]. Using this platform, participants’ responses at each time were documented with a unique, anonymous identifier that linked to their phone number. The Formr platform was programmed to send reminder SMS text messages 5 times a day. The SMS text messages contained the link for them to document their symptoms. This pilot intervention did not have cointerventions.

The design of this RCT followed the necessary procedures that are required for RFCBT. That is, to complete the RCT, participants were asked to self-report and document their ruminative episodes every 3 hours (4-5 times a day). Each time, if a participant documented their recent ruminative episode in the past 3 hours, they were prompted to document the trigger, immediate environments, duration, and emotional experiences related to the current ruminative episode. The intervention system was designed to be tailored to participants’ ruminative patterns in three ways: (1) based on participants’ ruminative episodes (triggers and immediate environments), participants would receive a set of training materials that best targeted the trigger of their ruminative episode (eg, problem-solving and interpersonal conflicts); (2) participants could choose to review their training materials at any time they wished as a means to provide in-the-moment feedback; and (3) participants could skip and withdraw from answering some of their documented prompts at a time when they were otherwise engaged in another activity. Therefore, each participant could have up to 175 times within the 35 days of the intervention to report on their ruminative episodes.

**Figure 2:**
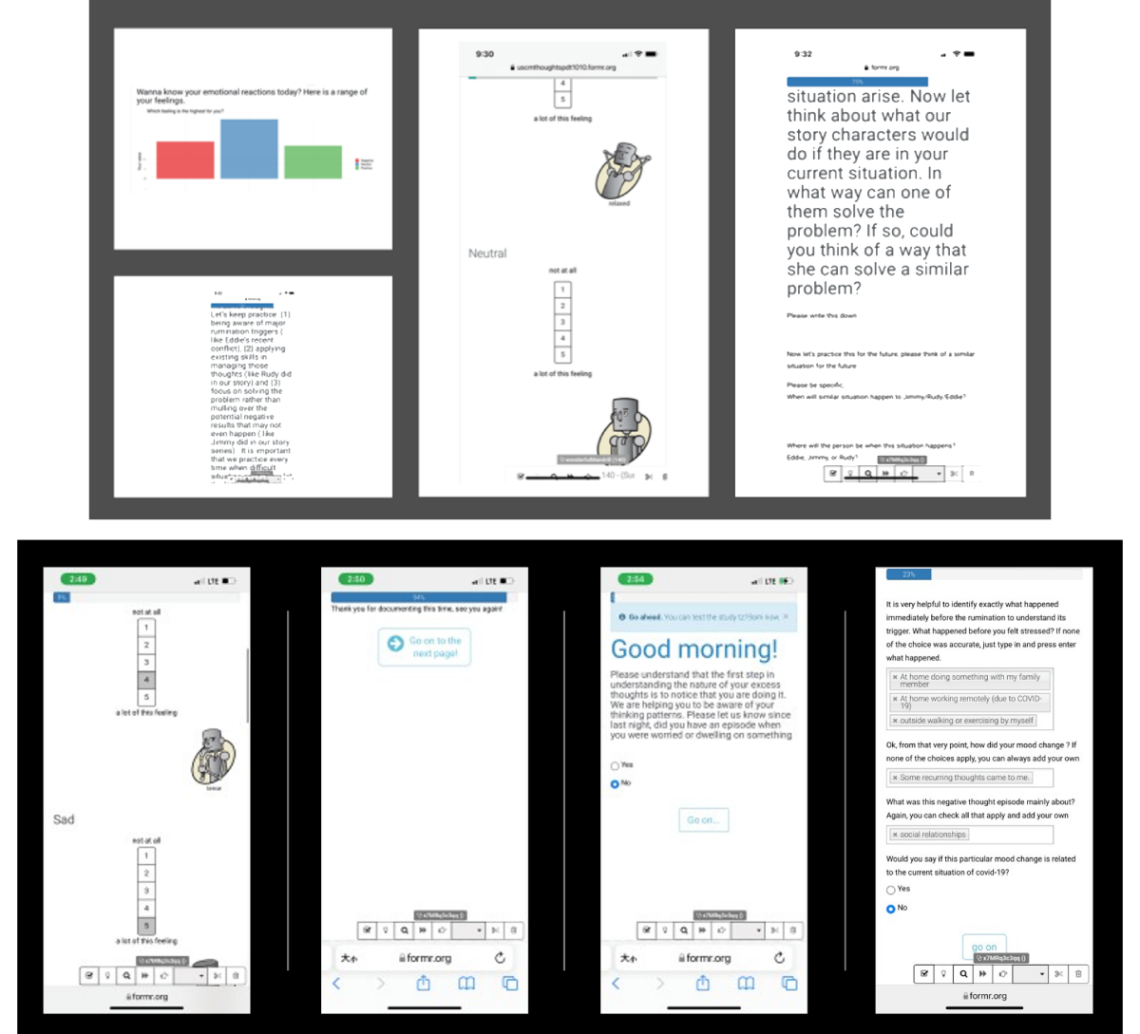
Randomized controlled trial platforms for participants to self-report and to receive information and feedbacks like the level of negative emotions one has experienced, and cognitive behavioral therapy training materials tailored to their current rumination episode. No-treatment control platforms for participants to self-report information (participants only report and document their rumination episodes.

To enhance our understanding of rumination and potential interventions to reduce it, participants were encouraged to document their ruminative episodes as many times as possible. Figure 2 shows the RCT platforms on a mobile phone screen.

### Ethical Considerations

This study was approved by the institutional review board at the University of Southern California (IRB; UP-20-01073-AM002). The IRB that reviewed this work required that, for this trial involving diagnosed patients with depression, researchers had to exclude patients who had not taken medication or had not visited a therapist in the past 3 months—we did so. Therefore, all participants in the treatment and control groups in this study were in active treatment for clinical depression. We explained to participants, both in the recruitment message and the consent form, that (1) the study was a double-blinded RCT to help reduce ruminative episodes related to depression, (2) there was an equal chance of being assigned to the no-treatment control and the JITAI condition, (3) this RCT was designed to test the effectiveness of mobile phones as a potential mode of delivery for rumination-based CBTs (MRFCBT) rather than to provide treatment for clinical depression, and (4) this RCT would only be delivered via mobile phones. They were also informed of the name of the research institution whose IRB approved the research. Participants were also entered into a lottery with 1 in 3 odds of receiving a US $50 gift card. A total of 59 volunteers agreed to be enrolled in this RCT and participated in the screener survey on the web.

### Recruitment

Participants in this study were recruited via a volunteering website originally developed using National Institutes of Health funds—ResearchMatch, a national web-based registry of >150,000 volunteers seeking to volunteer for new, potentially disorder-relevant studies. Upon approval from the IRB, ResearchMatch allows researchers to access and contact volunteers who are considering participating in research studies or clinical trials [40,41]. ResearchMatch offers filters for researchers to screen participants who may be eligible. It also has a message system for researchers to contact potential participants. We contacted potential participants who (1) were aged ≥18 years; (2) had self-reported recently (within the past 12 months) having been given a diagnosis of clinical depression; (3) had concurrently self-reported that they had no diagnosis of any other mental health disorder; (4) self-reported that they could understand, read, and write in English; and (5) reported that they had a smartphone with a data plan. Participants were also asked to indicate their medication and specify their most recent visit to a therapist. These were our inclusion criteria.

Of the 59 volunteers, 25 (42%) were eligible and provided their phone numbers. A total of 20% (5/25) of the volunteers never replied to the SMS text message reminding them to start the RCT. Of the 20 participants who logged on to the RCT website and initiated the RCT, 2 (10%) requested to quit the RCT during the first week, and 18 (90%) completed the RCT (ie, finishing 80% of the questionnaires and training tasks). On average, those 18 participants answered 145.76 (SD 13.35) out of 175 survey prompts, affording enough data points to conduct analysis on intensive within-person changes. Data for the 10% (2/20) of participants who left the RCT before the actual treatment (second week) were not included in the analysis. Table 1 shows the demographic backgrounds and other information of that 90% (18/20) of participants. A total of 50% (9/18) of the participants were assigned to the JITAI condition, and the other 50% (9/18) were assigned to the no-treatment control condition.

**Table 1:** Participants’ demographics and diagnosis information (N=18).

Characteristics		Participants, n (%)
**Gender**		
	Man	6 (33)
	Woman	12 (67)
	Nonbinary	0
**Income (US $)**		
	<30,000	3 (17)
	30,000-59,999	6 (33)
	60,000-89,999	7 (39)
	≥90,000	2 (11)
**Ethnicity**		
	Non-Hispanic White	11 (61)
	Hispanic	3 (17)
	Black	2 (11)
	Pacific Islander	0 (0)
	Native American	1 (6)
	Middle Eastern	1 (6)
	Asian American	0 (0)
**Depression diagnosis**		
	Major depressive disorder	12 (67)
	Persistent depressive disorder	1 (6)
	Psychotic depression	2 (11)
	“Situational” depression	1 (6)
	Mild depressive diagnosis	2 (11)

Double-blind assignment of participants was achieved through 2 survey platforms: participants first filled in a consent form and a screening survey on REDCap (Research Electronic Data Capture; Vanderbilt University), and REDCap automatically directed those who were eligible to the RCT survey platform, which randomly assigned participants to one of the 2 RCT conditions. There was no way to link the screener and contact information of REDCap to the RCT website. Therefore, neither researchers nor participants had access to RCT assignment information during the RCT. After participants completed the RCT, the researchers could retrieve the no-treatment control and treatment information per participant through website logs. There were no significant differences in demographics and diagnosis information by condition, as would be expected given randomization to condition.

Might participants have ascertained the condition to which they were assigned? This is possible. Unfortunately, we did not ask participants at the conclusion of the study what condition they thought they had been assigned to, and this should be added in future research. However, participants were unlikely to ascertain with any certainty the condition information. For example, participants in one of the experimental conditions might have thought they were in an experimental condition (vs control). However, it would have been hard to know if that experimental condition was not actually a control condition against some other experimental intervention group. Participants in the control condition also received message prompts every 3 hours to document their rumination experiences and emotional states. Therefore, participants who were assigned to the control group might have thought that they were in an experimental group, that is, an experimental group in which there was frequent monitoring of their rumination (eg, vs a no-monitoring control). Indeed, before the beginning of participation, although participants were told that there was an “experimental” and a “control group,” the nature of these conditions was not specified.

### Measurements

#### Overview

In addition to a set of demographic questions, we asked participants to document daily their level of COVID-19 stress and the total amount of standard drinks that they had consumed. To evaluate the ruminative experiences, participants also indicated their responses on a battery of depression measurements (see the following sections). After the intervention, participants were also asked to indicate their user experiences (see the following sections).

#### Ruminative Response Scale

Rumination patterns for each individual (and the differences therein) toward their depressive feelings were measured using the Ruminative Response Scale [42]. The Ruminative Response Scale consists of 22 items for assessing ruminative patterns and their dimensions (ie, depression, brooding, and reflection). This is a trait-like assessment of persistent rumination responses. In this study, participants rated each of these items on a scale of 1 to 7 (1=not at all like me and 7=extremely like me), such as “I think about how alone I feel” or “what am I doing to deserve this?” This measure exhibited excellent reliability in this study (Cronbach α=.95).

#### Ruminative Episode and Duration

To evaluate participants’ experiences of rumination, we asked 2 questions. First, *in the past 3 hours, did you experience a ruminative episode (by ruminative episode, we mean experiencing a battery of negative thoughts that distracted from your current situation)?* Second, if the participants answered “yes,” they were asked, “how long did this ruminative episode last?”

#### State Depression Scale

The State Depression Scale (SDS) is a 3-item scale for participants to rate their in-the-moment level of depressed mood, anhedonia, and irritability on 7-point Likert scales ranging from 1 (not at all) to 7 (very much) at the time of the alert. The SDS has already been tested and used in previous EMA studies on rumination and depression [43]. This scale was presented as a slider bar to fit the participants’ mobile phone screen size. The Cronbach α for this scale has consistently been high (ie, .8 to .9 range) each time it is used in research.

#### Emotional Experiences

Participants’ in-the-moment emotions at the time of sampling were measured using a pictorial self-report by the name of Pick-A-Mood. This is essentially a 7-point Likert scale measuring a range of feelings such as tense, annoyed, calm, and relaxed. Pick-A-Mood was designed specifically for mobile use [44], which serves as a better alternative than other emotion measurements. The 8 emotional items were later combined into a set of positive, neutral, and negative feelings. As in the case with the SDS, the Cronbach α for this scale was consistently high (ie, .8 to .9).

### Statistical Analysis

#### Power

As the goal of this RCT involved evaluating changes in ruminative episodes during the intervention, it follows the recommended design of an intensive within-person, microrandomized trial. There are 2 issues pertaining to power that are important to differentiate. First, normally when considering power for interventions, the authors calculate the number of participants needed for sufficient power (eg, 0.80) to detect an effect given one’s design. Unfortunately, there is currently no concrete standard to calculate this type of sample size for adequate power (or power given sample size) in such trials to formally test the effectiveness of the intervention versus the control group per se [45]. There is a second type of power consideration that is specific to Group Iterative Multiple Model Estimation (GIMME). The issue is as follows: are there enough data points (eg, EMA events) within participants for the researchers conducting such analyses to trust the rigor of the results? That is, with GIMME, to have sufficient power to detect a small effect size when researchers are tracking individual changes, each participant is required to document changes in their behaviors of interest with sufficient time points (approximately n=60-75) [45]. Moreover, GIMME package developers note that it usually takes 7 to 10 participants within each comparison group to detect between-group differences [46,47]. In this study, of the 18 participants who completed the study, 9 (50%) were in the control condition and 9 (50%) were in the JITAI condition.

#### The GIMME Approach

Today’s technologies (eg, smartphone just-in-time interventions) can provide an enormous amount of intensive data over time for a given person. At any given point in time when given events occur (or not), an individual may have co-occurring affective reactions, cognitions, and other states. In addition, each or none of these phenomena in the moment (and their relationships with one another) may predict reactions at the next moment in time (and the relationship among elements at that later point in time). Often, these complex data are merely averaged over time or over chunks of time within a person, with considerable loss of information as a given individual moves from moment to moment and context to context, where variability in behavior at the within-person level needs to be understood. In our work, for example, we would argue that a therapeutic client may benefit more from a rumination intervention if given within a few moments of a potentially causal event, cognition, or emotional reaction that precipitated rumination than from an intervention that happens the next day or a week later outside the current context. Such detailed co-occurrent and cross–time-lagged networks of information are critical for therapists who would desire to know when to provide what therapeutic message to optimize their effectiveness. To be able to understand this intensive within-person idiographic level of response and respond to it, we need newer statistical approaches.

The need for intensive within-person measurement has already become apparent in other areas of social science using intensive (every 3 hours) EMAs, such as in personality psychology. Over the last 10 years—recognizing the importance of within-person variability that cannot be accounted for based on between-person measures (eg, Big Five trait measures)—the field has embraced the importance of GIMME, which affords an intensive within-person assessment over time. Beck and Jackson [48], publishing in the flagship journal of the field, the *Journal of Personality and Social Psychology*, delineated this new approach to idiographic personality models and how it identifies varying patterns of behavior and behavior change by person by context and lagging over time over 2 years. The method affords insight compared with nomothetic approaches but also compared with other idiographic approaches that only look at phenomena at one or relatively few points in time. GIMME affords an incredible tool for examining these sophisticated within-person dynamics over context and time.

Although GIMME is a relatively new approach, it leverages many existing statistical approaches that have been widely used for many decades across science. For example, GIMME leverages network science (and computer science) approaches to implementing network theory. Although a full description of this approach is beyond the scope of this paper, the statistical rationale for network analysis has been delineated in leading journals in science, including in *Nature* (eg, see the work by Whitfield [49]). In brief, network theory is a part of graph theory [50]—a network can be defined as a graph consisting of attributes such as nodes (eg, in this case, rumination episodes and affective states) or edges (associations between nodes). Therefore, the edges in a network (as in GIMME) are essentially correlation coefficients. GIMME allows us to examine the relationships (symmetrical or asymmetrical) among these attributes in a network cotemporaneously as well as lagged over time. GIMME takes advantage of the recent advances in idiographic models and network science. Rather than using a nomothetic multivariate time series analysis, GIMME uses a network approach that highlights relationships among variables both visually and quantitatively, representing both direct and indirect associations among them. Beck and Jackson [48] delineated how idiographic networks can answer questions involving intraindividual change that are not easily addressed using nomothetic models [51].

At the within level, GIMME uses an estimation analogous to autoregression. Using GIMME, one can model the subsequent scores (or occurrences) of an event using the previous scores (or occurrences). For example, in this study, we measured every 3 to 5 hours whether a participant experienced rumination in each of the 35 days of the study period. We examined contemporaneous and lagged relationships. Contemporaneous relationships estimate probabilistic within-person relationships at the same time point. This is the tendency for ruminative symptoms to occur at the same time. Lagged relationships examine probabilistic within-person, cross–time-point (or cross-lagged) relationships. This is the tendency for ruminative symptoms to follow one another across measurement conditions. For example, in general, for ruminative behavior, if a rumination episode happens at time 1, then there is a high possibility for a similar rumination episode to happen later. Using those experienced rumination episodes, one can use GIMME to construct a time series model of subsequent occurrences of rumination episodes in the same way that one can construct a time series multilevel model, but one can construct a model that is truly ideographic as there is no shrinkage of individual-level estimates [52]. That is, GIMME can model the associations between co-occurring scores (eg, symptoms or occurrences) both concurrently (0 lag) and at cross–time lags. In our analysis, we also measured every 3 to 5 hours scores of affective reactions of participants. Putting scores of affective reactions as co-occurring variables with rumination, GIMME can generate a model to estimate both (1) the association between affective reactions and rumination at the same time when they occur and (2) whether previous affective reactions could be predictive of other variables in the model. In this study, we used GIMME to examine whether previous affective states could be predictive of the occurrence of subsequent rumination episodes. Thus, GIMME [52,53] can reliably obtain the presence and direction of effects among variables collected at multiple (intensive) time points, and it uses the full information maximum likelihood that rigorously estimates the missing data points.

Furthermore, GIMME estimates both between- and within-person effects similar to a multilevel model, where there is dependence between individual- and group-level estimates. Specifically, GIMME can estimate both associations among variables measured simultaneously and how variables measured at a particular time point predict the effects and directions of relationships with variables collected at subsequent time points. To do so, GIMME estimates those associations and directions of effects through both unified structural equation modeling (uSEM) and network analysis. That is, in estimating a GIMME model, the algorithm first uses uSEM, a structural vector autoregressive model, to fit both the contemporaneous and lagged parameters. However, it does so in a way that is different from a multilevel model that simply estimates “average” relationships—GIMME keeps group-level paths that are significant for at least 75% of individuals and gets rid of paths that are not significant for at least 75% of the sample. GIMME uses the selected path from uSEM to retain those group-level paths [46,54].

GIMME models work well for considering the effects of an intervention—participants who reported experiencing a rumination episode were immediately sent a JITAI message tailored to the specific individual trigger of their rumination (eg, problem-solving skills or interpersonal skills). The GIMME model, with its capacity to evaluate the significant changes in rumination episodes since their previous occurrences, can evaluate the extent to which tailored message feedback was successful in helping participants take control of their condition by showing whether there was a significant reduction in rumination episodes from their previous rumination occurrences. After fitting each individual model, GIMME also fits a group- or subgroup-level model that looks at between-group effects. GIMME analysis also produces network connections for each participant and group (if there is a group-level effect). Network graphs are produced by treating each variable of interest as a node and the associations (both contemporaneous and lagged) between each variable as edges [46,54]. Analyses were conducted using the GIMME package in R [46,54].

## Results

### Overview

**Figure 3:**
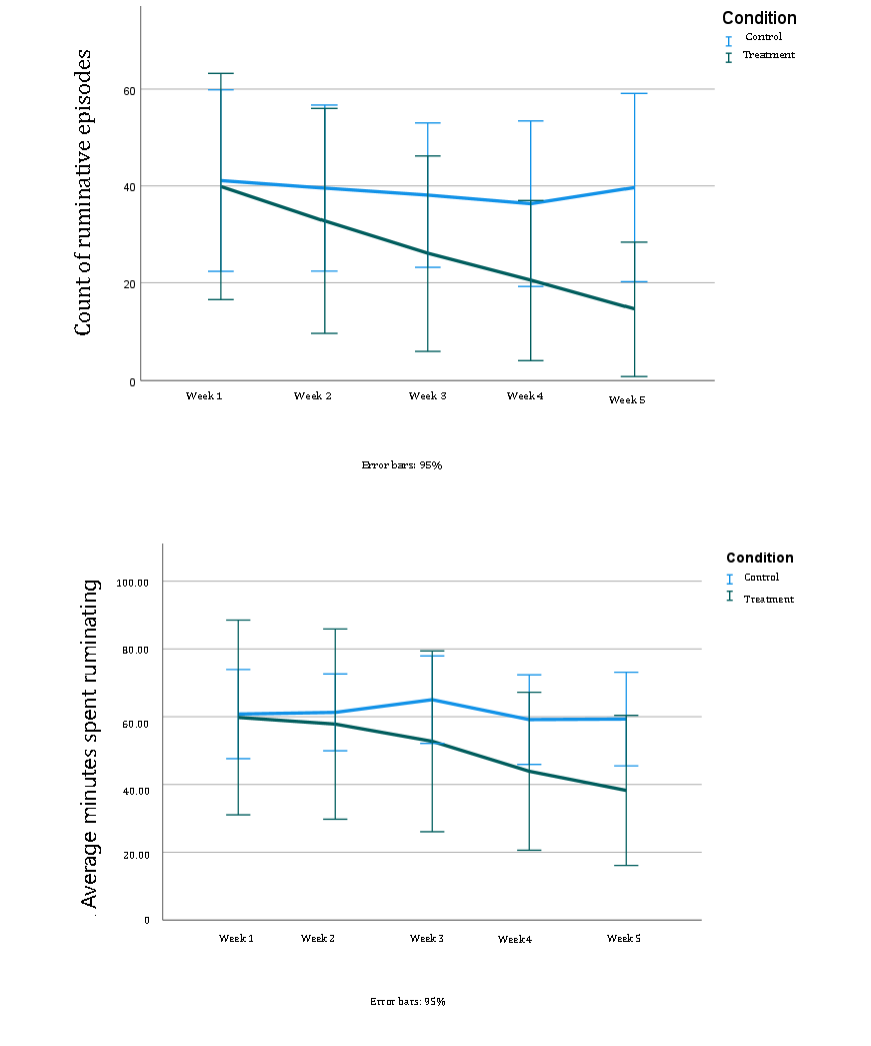
Changes in count of ruminative episodes and average minutes per each ruminative episode 5 weeks.

Hypothesis 1 predicted stronger effects of the JITAI-MRFCBT condition versus the control condition in reducing depressive rumination. Table 2 shows each participant’s reported statistics (ie, total count and average duration) regarding ruminative episodes (“in the past three hours, have you experienced a negative thinking episode?”). Table 2 is a summary of rumination episodes and average minutes spent per rumination episode per week throughout the 5-week period. Owing to the limited sample size (each group had 9 participants), we used bootstrapped independent 2-tailed *t* tests to compare differences between the treatment and control conditions for the baseline and postintervention weeks. In the baseline week (Table 2), there were no significant differences in the counts of rumination episodes (t_16_=−0.94; *P*=.93) and the average time spent ruminating between the treatment and control groups (t_16_=−0.73; *P*=.94). We also calculated reduction values. Reduction of rumination episode counts and reduction in time spent on rumination were created by subtracting rumination episode counts in week 5 from those in week 1 per participant**.** Between groups, using a series of independent *t* tests with bootstrapping, results showed that participants in treatment (mean −25.28, SD 14.50) reported a significantly greater reduction in rumination episode counts than those in the control condition (mean 1.44, SD 4.12, 95% CI 14.25-33.67; *P*<.001; Cohen *d*=2.5). Similarly, those in the treatment condition (mean −21.53, SD 17.6) showed a significant reduction in average minutes spent in rumination compared with those in the control condition (mean 1.47, SD 1.5, 95% CI 10.58-32.00; *P*=.04; Cohen *d*=1.84). Figure 3 also shows participants’ counts of ruminative episodes and the average time they spent on each rumination episode per week.

**Table 2:** The count of rumination episodes and average duration (in minutes) of each ruminative episode each week.

Condition and participant ID			Week 1, N^a^		Week 2, N		Week 3, N		Week 4, N		Week 5, N		Week 1, mean (SD)^b^		Week 2, mean (SD)		Week 3, mean (SD)		Week 4, mean (SD)		Week 5, mean (SD)
**Treatment**																					
	i_1	20		19		7		11		9.5		18.75		17.964		16.53		13.24		14.2308	
	i_2	5		1		0		0		0		15		15.6		10.01		5		0	
	i_3	25		7		4		3		2		113.571		107.56		100.3		60.7		56.5	
	i_4	97		89		69		53		54		55.55		50.5		45.5		40.5		35.4	
	i_5	50		38		34		27		20		110		110.6		97.6		89.5		79	
	i_6	15		15		7		4		3		20.45		20.5		15.89		9.76		9.4	
	i_7	29		12		11		5		0		55.9		50.89		45.3		37.8		21.4	
	i_8	40		44		43		27		13		69		68.33		67.89		65.3		56.2	
	i_9	78		70		60		55		30		79.99		78.63		75.8		73.46		72.3	
**Control**																					
	i_10	98		88		78		80		99		75		70		78.8		78.9		75.2	
	i_11	30		22		25		28		29		55		55.7		79.3		56.35		53.5	
	i_12	45		44		43		42		44		56.5		65.5		56.3		54.5		53.5	
	i_13	42		41		43		42		41		62.5		62.5		63.52		45.5		60.25	
	i_14	19		18		15		16		21		89		85.3		86.5		85.5		87	
	i_15	23		22		24		20		22		43		46.3		50.36		43.5		40.47	
	i_16	32		33		35		12		20		31.23		33.73		32.3		33.23		28	
	i_17	56		59		55		60		55		69.68		67.5		68.6		69.7		70	
	i_18	25		29		25		27		26		65.3		65.12		69.7		65.3		66.04	

^a^Count of ruminative episodes.

^b^Average minutes spent on rumination.

### Principal GIMME Results

GIMME provides a detailed look at the rumination reduction found in this study. This is because traditional *t* tests could not provide a rigorous estimation for RCT effects because of the sample size at the between-group level [51]. A GIMME analysis was conducted to evaluate within-person changes, which provided robust results in explaining the effects of this RCT. We specified the analysis as a multiple group estimation that compares the differences between the JITAI MRFCBT and no-treatment control conditions. GIMME modeling first estimates the association between each variable as β coefficients at each individual level (if any). Table 3 shows a set of fit indexes that indicate the model fit for each within-person change model (18 individuals) using the uSEM approach. The root mean square error of approximation (RMSEA) was evaluated with chi-square tests. In this case, an RMSEA of 0.01 is considered an excellent fit [55]. A measure of comparative fit is the comparative fit index (CFI); in this case, CFI=0.98. Hu and Bentler [56] suggested that RMSEA values <0.06 and CFI values >0.95 are indicative of a good fit. A more recent measure of fit to avoid model misspecification is the standardized root mean square residual (SRMR) [57]. In this case, an SRMR of 0.06 indicates a good fit as it is <0.08. Our results show that a great majority (15/18, 83%) of these models achieved excellent fit. The estimation of group-level effects is set at 75%, meaning that group-level analysis will not be conducted unless 75% of the participants have shown an excellent fit [46,54].

**Table 3:** Within-individual fit indexes in Group Iterative Multiple Model Estimation (GIMME; waitlist control and just-in-time adaptive intervention [JITAI] comparison).^a^

Condition and participant ID			Chi-square (*df*)		*P* value		RMSEA^b^		SRMR^c^		NFI^d^		CFI^e^
**JITAI condition**													
	individual_1	109.9389 (78)		.01		0.07		0.07		0.96		0.97	
	individual_2	101.2896 (77)		.03		0.06		0.07		0.96		0.97	
	individual_3	97.9413 (79)		.07		0.07		0.09		0.97		0.98	
	individual_4	94.4551 (79)		.11		0.05		0.06		0.98		0.99	
	individual_5	123.9945 (77)		<.001		0.07		0.05		0.95		0.97	
	individual_6	111.9069 (80)		.02		0.06		0.07		0.95		0.97	
	individual_7	114.0346 (79)		.36		0.02		0.06		1.00		1.00	
	individual_8	116.1622 (76)		.02		0.07		0.06		0.96		0.97	
	individual_9	118.2899 (74)		<.001		0.09		0.05		0.94		0.96	
**Control condition**													
	individual_10	120.4176 (79)		<.001		0.40		0.05		0.95		0.97	
	individual_11	122.5452 (76)		.04		0.30		0.05		0.94		0.97	
	individual_12	124.6729 (76)		.08		0.35		0.05		0.94		0.96	
	individual_13	126.8006 (76)		<.001		0.45		0.05		0.94		0.96	
	individual_14	128.9283 (75)		.02		0.52		0.05		0.94		0.96	
	individual_15	131.0559 (75)		.02		0.59		0.04		0.94		0.96	
	individual_16	95.5461 (77)		.01		0.05		0.06		0.98		0.99	
	individual_17	85.0285 (76)		.22		0.05		0.08		0.96		0.98	
	individual_18	146.7335 (77)		<.001		0.11		0.05		0.94		0.96	

^a^Fit indexes provided by GIMME are subject to the same rule of thumb in structural equation modeling [46].

^b^RMSEA: root mean square error of approximation.

^c^SRMR: standardized root mean square residual.

^d^NFI: normed fit index.

^e^CFI: comparative fit index.

A sample of β correlations per participant (individual 16 in the control condition and individual 7 in the JITAI condition) showing predictive models based on a participant’s self-reported rumination relationships can be found in Tables 4 and 5, respectively (we did not provide the full estimation for all 18 participants to avoid repetition). The results (Table 4 for individual 16) showed that the rumination episode significantly predicted the occurrence of a subsequent rumination episode (β=.022; *P*=.02). Table 5 shows a model that describes another participant’s (JITAI condition, individual 7) self-reported changes during the intervention. This model also shows an acceptable model fit (Table 3), with RMSEA=0.02, SRMR=0.06, and CFI=0.98. Interestingly, we see that, for this participant, the rumination episode (episode) was not a significant predictor of the occurrence of a subsequent rumination episode (β=−.03; *P*=.75).

**Table 4:** Sample path analysis within individual 16 (control condition).^a^

Predictor	DV^b^	β	SE	*z* score	*P* value
Episode^c^	Episodelag^d^	.022	0.09	2.43	.02
Durationmins^e^	Durationminslag	.02	0.06	0.37	.71
StateRM^f^	StateRMlag	−.06	0.04	−1.41	.16
Neutral^g^	Neutrallag	−.11	0.10	−1.11	.27
Negative^h^	Negativelag	.24	0.09	2.52	.01
Positive	Positivelag	.12	0.08	1.40	.16
Covidst^i^	Covidstlag	.91	0.02	52.88	<.001
Drinks^j^	Drinkslag	.86	0.03	33.58	<.001
StateRM	Episode	1.17	0.06	20.82	<.001
Durationmins	Episode	.78	0.04	19.50	<.001
Episode	Neutral	−.40	0.08	−4.73	<.001
Negative	StateRM	.30	0.09	3.23	<.001
StateRM	Durationmins	−.35	0.07	−4.85	<.001
Positive	Negative	−.44	0.07	−5.87	<.001

^a^*Episode* is the occurrence of a ruminative episode at each time; *stateRM* is the state depressive rumination level; and *negative*, *positive*, and *neutral* indicate participants’ emotional state.

^b^DV: dependent variable.

^c^Episode: the number of rumination episodes.

^d^The affix *-lag* that comes after each variable (eg, episodelag or positivelag) indicates the subsequent scores and occurrences of the variable.

^e^Durationmins: minutes spent on a rumination episode.

^f^StateRM: level of rumination at the rumination episode.

^g^Neutral: level of neutral affect in the past 3 to 5 hours.

^h^Negative: level of negative affect in the past hour.

^i^Covidst: stress related to COVID-19.

^j^Drinks: drinking related to rumination.

**Table 5:** Sample path analysis within individual 7 (just-in-time adaptive intervention mobile rumination-focused cognitive behavioral therapy condition).^a^

Predictor	DV^b^	β	SE	*z* score	*P* value
Episode^c^	Episodelag^d^	−.03	0.09	−0.32	.75
Durationmins^e^	Durationminslag	.00	0.09	0.00	>.99
StateRM^f^	StateRMlag	.01	0.04	0.16	.87
Neutral^g^	Neutrallag	−.08	0.09	−0.94	.35
Negative^h^	Negativelag	.18	0.08	2.23	.03
Positive	Positivelag	.03	0.11	0.23	.82
Covidst^i^	Covidstlag	.96	0.01	98.47	<.001
Drinks^j^	Drinkslag	.99	0.00	625.20	<.001
StateRM	Episode	.73	0.05	14.79	<.001
Durationmins	Episode	.59	0.07	7.99	<.001
Episode	Neutral	−.64	0.07	−9.78	<.001
Negative	Staterm	.03	0.12	0.26	.79
Neutral	Positive	.60	0.07	8.55	<.001
StateRM	Neutral	−.26	0.06	−4.61	<.001
Negative	Neutral	−.64	0.11	−5.77	<.001

^a^*Episode* is the occurrence of a ruminative episode at each time; *stateRM* is the state depressive rumination level; and *negative*, *positive*, and *neutral* indicate participants’ emotional state.

^b^DV: dependent variable.

^c^Episode: the number of rumination episodes.

^d^The affix *-lag* that comes after each variable (eg, episodelag or positivelag) indicates the subsequent scores or occurrences of the variable.

^e^Durationmins: minutes spent on a rumination episode.

^f^StateRM: level of rumination at the rumination episode.

^g^Neutral: level of neutral affect in the past 3 to 5 hours.

^h^Negative: level of negative affect in the past hour.

^i^Covidst: stress related to COVID-19.

^j^Drinks: drinking related to rumination.

**Figure 4:**
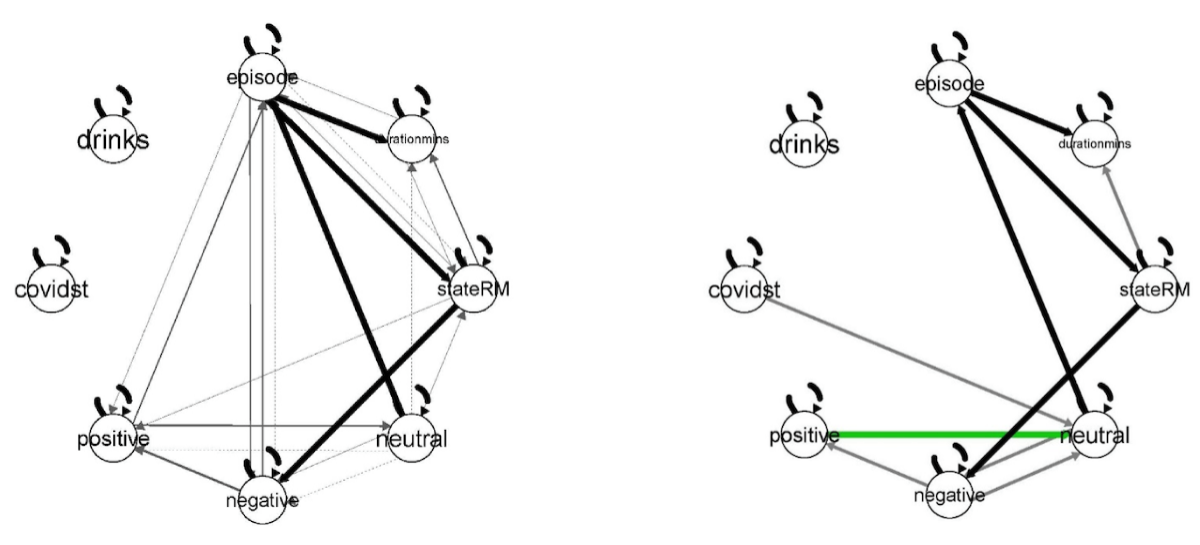
Between-group effects (no-treatment control vs just-in-time adaptive intervention [JITAI] mobile rumination-focused cognitive behavioral therapy [MRFCBT]). Note: the graph on the left shows the associations of different variables within the no-treatment control, while the one on the right shows the overall path models within the JITAI MRFCBT condition. Solid lines indicate a contemporaneous effect; dashed lines indicate a lagged effect. Black lines indicate a group-level effect; green paths indicate an effect at a subgroup; gray paths indicate an individual effect.

The GIMME results of comparisons both at the group (Figure 4) and individual levels provide a detailed look at the network of associations and effect carryover in time. In this analysis, shown in Figure 4, our model showed that, at the between-group level, there are significant predictors that are responsible for the occurrence of a subsequent ruminative episode. For example, in the control condition, we found that factors such as rumination episode, negative emotions, and duration of the rumination episode were also estimated as significant predictors of a subsequent rumination episode. In Figure 4, for the JITAI-MRFCBT overall effects, it is noteworthy that the dashed lines found in the no-treatment control are absent, suggesting little carryover from earlier rumination episodes and their duration. The other noteworthy difference between these conditions is the presence of a green line from positive to neutral. This can be interpreted as the presence in the moment of more positive affect that may be bolstering the user’s ability to respond more productively in the face of the rumination episode.

The lack of dashed lines for participants in the treatment group indicates that these participants did not seem to generate as many subsequent cascading rumination episodes compared with the control participants. This difference at the group level and the individual level—and the detail with which we can “see” these network processes over time—suggests that participants in the JITAI condition did not bring their negative thoughts and previous ruminative experiences into their next ruminative episode. Indeed, the main goal of the JITAI-MRFCBT condition was to train participants to make different attributions and focus them on problem-solving when they experienced a particular ruminative episode.

## Discussion

### Principal Findings

This pilot study compared a JITAI-MRFCBT condition with a no-treatment control condition in reducing rumination. To assess rumination reduction, we compared the baseline (week 1) and the postintervention week (week 5)—we found that both ruminative episodes and the average time participants spent on those ruminative episodes decreased significantly in both groups, but the reduction was significantly greater in the JITAI-MRFCBT condition. Our results showed that participants in the control condition demonstrated some time-lagged effects such that experiencing ruminative episodes or negative emotions at a time point predicted an increase in such experiences subsequently. However, such lagged effects were not present in the JITAI condition. That is, no dotted lines in the treatment condition indicate lagged effects. This difference at the group level shows that participants in the JITAI condition, compared with those in the control condition, did not bring their negative thoughts and previous ruminative experiences into their next ruminative episode. The treatment appeared to stop the “echo chamber” of the previous negative experiences.

This study, in addition to affording a comparison between treatment and control groups at both the group and the individual level, involved four innovations: (1) using MRFCBT as the “what” of the intervention and JITAI as the “when,” which has promise for optimizing responsive and effective interventions per client; (2) devising a “how” (in this case using participant baseline measures of rumination and GIMME) to identify typical baseline patterns per participant when rumination (and change in affect and cognitions) is likely; (3) using GIMME within persons per client to better understand “why,” leveraging smartphone technologies to deeply understand and predict, for the first time, networks of cognitions and affect and patterns of rumination and cascading rumination (and possible interpersonal predictors) from one moment to the next; and (4) ability (via combining these technologies) to afford identification, understanding, and change in disruptive psychological patterns (eg, rumination) personalized to the user leveraging smartphone technologies. In the following sections, we discuss each of these innovations along with their implications for the literature on rumination, depression, interpersonal communication, and techniques and technologies (eg, GIMME, EMA, and JITAI). We then discuss the limitations of this work and our overall conclusions.

### Comparison With Prior Work

#### The “What” and “When” of CBT Using JITAIs

CBT has been a “gold standard” for a diverse array of disorders. The emergence of mobile phones as an alternative means to deliver face-to-face CBTs also speaks to an increasing need for mental health services. Researchers have been working on phone apps [58,59] to provide CBT (ie, MCBT) to those who are depressed. Despite reports of positive user experiences [60], there is still a lack of symptom-focused MCBTs (eg, MCBTs for depressive rumination). MCBTs to date, unfortunately, still lack the level of interactive features needed to provide more tailored feedback based on individual characteristics. This is despite calls to address this need [60].

However, for many disorders, CBT delivered face-to-face is apt to be less than optimally effective as clients cannot receive the treatment at the optimal time. Even when included in a mobile device, CBT is typically not responsive to the dynamics of the client’s experience. Rumination provides a very good example of these difficulties. If negative cognitions and affective states are allowed to cascade, as mentioned earlier, this exacerbates depression. Simply providing CBT materials people can access even on a mobile device is likely not enough. A scaffolding tool for responding at just the right moment with the right therapeutic tools and messages for mitigating rumination threats should be the intervention goal to which we aspire. Although no previous study has tested mobile phone–delivered JITAIs in reducing depressive rumination [26], the results of this study suggest that JITAIs could afford an essential means to deliver more responsive CBT for ruminating clients diagnosed with clinical levels of depression and that, in doing so, especially with intensive rumination measures and treatments responsive to them, rumination could be reduced.

Interesting transdiagnostic CBT approaches are promising treatments for a broad range of emotional disorders but, in reviewing this work, only rumination and negative metacognition significantly mediated client functioning [61]. These findings highlight the importance of mitigating the effects of rumination for the effectiveness of CBT interventions, thus highlighting the importance of this method and approach for “seeing” and reducing rumination.

#### The “How” of JITAIs for MCBT

JITAIs focus on the “when” of intervention delivery [27]. However, knowing “when” to deliver a given intervention—the “what” (eg, MCBT component)—depends on what cues and indicators are available and should be used (eg, with one’s smartphone) to make that determination. For example, JITAIs are used in trying to inhibit an individual’s alcohol [62] or illicit drug use [63,64], which need interventions to note a client’s typical source of these substances (eg, local bars and location of dealers). Knowing what environmental or personal cues might initiate a problematic behavior (that the intervention is designed to deter) provides the basis for the “how” (how JITAIs know “when” to intervene). Some possibilities are through EMA and sensor technologies in the smartphone—these can potentially provide the basis (ie, possible algorithms based on formative research) for a JITAI to “know” “when” to deliver “what” to optimize intervention goals. In this study, rumination had long been identified as problematic in exacerbating depression [31,33,37]. The “how” for JITAI in this study became how can we determine when a client (who ruminates and is diagnosed with depression) is most likely to be ruminating? Our interim step in ascertaining a relatively crude “how” was to first determine how often individuals who were clinically depressed (and ruminating) ruminated, for example, how many times a day did this occur? Across individuals in this population, there was variability in these patterns. Therefore, “how” required a more personalized assessment (ie, a baseline) of the times of day. The goal of RFCBT is to train participants to make different attributions and focus on problem-solving when they experience a particular ruminative episode. The presumption is that, when the next ruminative episode occurs, participants can immediately become aware of their ruminative thoughts and adopt one of the strategies offered to them by RFCBT. In this study, we achieved this goal through the week 1 baseline assessment. That is, we used the individualized information, which tells us the frequency, duration, types, and high time of each client’s rumination patterns, to create messages tailored to each participant. For example, if the week 1 baseline information told us that a participant had been experiencing more rumination episodes elicited by interpersonal stress rather than personal setbacks, this participant received more messages on interpersonal problem-solving.

Our process of using baseline EMA reports for the “how” for estimating probable rumination times provides a model not only for “rumination” estimates at the individual level but also for any behavior of interest we seek to change. That is, can we identify in time and space when this behavior of interest is more probable per person of interest? Can we identify cues or indicators (and possible algorithms using smartphones and EMA) that anticipate the behavior of interest? Can we collect that information at baseline to then use it in the intervention of a personalized JITAI?

### Sufficiently Fine-Grained Measurement for the “Why”: Blocking Rumination Cascades

The reported findings using GIMME within persons per client provide insight into the “why” of ruminative cascades. Identifying rumination, rumination cascades, and rumination blocking is a known challenge [3,65]. The emotional cascade model suggests that emotional dysregulation turns into subsequent behavioral dysregulation through a process called emotional cascades [66]. Emotional cascades are the feedback loops between intense rumination and negative affect. Emotional cascades occur mostly after a negative trigger such as unpleasant interpersonal experiences, personal losses, and setbacks. As one becomes more focused on the negative experiences, one can find it hard to divert attention away from reliving the negative event. This cycle is repeated as rumination and negative affect interact, resulting in more dysregulated behaviors. However, “seeing” these networks of cognition and affect, especially in anything close to “real time,” has not been possible. This makes it hard to understand what exactly is triggering in the situation for this individual and how it is being framed by them. Such insights could provide targets for personalized interventions. GIMME provides a method for examining at the individual level the associations between cognition and affect for the individual at each time point and how these associations carry over and predict subsequent ruminative episodes. GIMME methods, and sufficient time sampling using EMA, afford tools to not only understand the client’s cognitive and affective construals (eg, activating hurtful perceptions) of interpersonal interactions but also “see” those with detail in a more fine-grained network than has been previously available. This makes it possible for researchers, clients, and their therapists to identify how these perceptions change and trigger rumination events for specific individuals with depression from moment to moment over time (and whether interventions designed to address them are proving effective).

Numerous empirical studies have highlighted the link between ruminative process and behavioral dysregulation and called for effective means of rumination disruption. However, research suggesting ways to effectively break this vicious cycle is scarce. Blocking rumination is historically difficult to achieve [3,65]. An approach is to use “normal methods of distraction,” which unfortunately “becomes ineffective” [65]. Part of the difficulty may be that, historically, rumination has been assessed through self-reports, often summarizing over days, weeks, or months. As far as we know, there are no current effective measures of rumination closer to “in the moment” in real time. The results of this study suggest that GIMME tools and personalized baselines can help us better “see” rumination in a more fine-grained way in each episode. This means that we can better “see” whether our interventions are effectively reducing the ruminative links from one ruminative episode to the next. “Seeing” in a fine-grained way when disruption of rumination is occurring and why is a major advance.

### Combining Intervention Design, Techniques, and Technologies: Advancing Rumination Science

A major innovation in this pilot work is (1) putting these 3 innovations together, leveraging MRFCBT materials to afford identification, understanding, and change in rumination patterns, all while personalizing the intervention to the user (when to intervene with what), leveraging smartphone technologies, and (2) showing the promise of this JITAI-MRFCBT compared with the no-treatment control group. JITAIs, as used in this study, are an effective method to disrupt emotional cascades rather than simply creating distraction—instead of using traditional means of shifting attention (eg, physical sensation seeking), participants can be provided with a scaffolding frame that helps them reappraise their current emotions and alter attributions at the exact moment of the emotional cascade.

In addition, this pilot trial evaluated the promise of a mobile phone–delivered JITAI (with MRFCBT) as a delivery means of reaching participants with an in-the-moment intervention [27,67]. The results of this project have shown that, in JITAIs, participants ruminating can receive in-the-moment feedback on their mobile phones. Third, this study provides a data-driven explanation regarding the effects of JITAIs. That is, our GIMME results suggest that, with messages timely tailored and sent to participants in the time of greatest need, participants in the treatment condition showed a significant reduction in “next-time” rumination episodes. Results from the JITAI conditions are promising for reducing the number of ruminative episodes and lowering average rumination times. Therefore, the results of this pilot RCT study indicate the viability of a full-scale RCT for reducing rumination in a population diagnosed with clinical depression. Thus, this pilot serves an overarching goal of identifying a viable approach that can deliver JITAIs focusing on depression outcomes and provides the groundwork for subsequent clinical trials.

Rumination plays a significant role in a wide array of mental health disorders and problematic behaviors. The focus in this study was on MDD, but rumination also plays a significant role in other mental health disorders such as bipolar disorder [68], anxiety, and mood disorders [66,69]. Nolen-Hoeksema [30], noting the high comorbidity among many mental health disorders, conceptualized rumination as a transdiagnostic process associated with not only depression and anxiety but also a variety of deleterious outcomes (ie, substance abuse, binge eating, and self-injuring behaviors; see McLaughlin et al [66]). Indeed, emerging literature provides evidence to support such claims [66,70,71], with McLaughlin and Nolen-Hoeksema [70] arguing for “the importance of targeting rumination in transdiagnostic treatment approaches for emotional disorders.” As recently argued, “there is consistent evidence showing that a reduced use of cognitive reappraisal and an increased use of negative rumination are present across a number of disorders, whereas increased levels of positive rumination appear to be confined to bipolar disorder” [72]. Given the transdiagnostic role of rumination across many disorders, the importance of tools to measure and understand it (and intervene with respect to it) in a detailed way for individual clients across contexts and over time is critical and highly generalizable.

### Limitations

However, the limitations of this RCT are apparent. The major limitation is the self-reported nature of participants’ depression diagnoses. Although we designed a series of questions such as asking participants to report their recent diagnosis and provide names of their depression medications, we may still not be able to fully generalize the efficacy of these interventions to all participants with clinical depression. Therefore, the results of this study are only limited to participants with self-reported depression diagnoses. There are other less major limitations. First, despite the small sample of participants, this analysis could offer a robust pre- and postestimation of the RCT following suggestions of a micro-RCT. However, this suggests a need for a full-scale RCT with power that can speak to between-group comparisons. Therefore, a subsequent RCT is needed. Second, because of delays in the IRB process of >1 year, some exploratory research questions on how COVID-19 and drinking patterns at the time of COVID-19 (and the impact on ruminative patterns) could not be examined in the timely way originally planned. Among participants who provided daily entries of their COVID-19–related stress, most (18/25, 72%) indicated that they experienced zero or very low COVID-19 stress. Therefore, we did not detect any significant role of COVID-19 and related drinking behaviors (shown in the GIMME models). That is, the results of this study could only be evaluated as a pilot for subsequent RCTs during future widespread events. Third, although this RCT showed some effects of treatment in reducing ruminative episodes among participants with clinical depression who were in therapy, it did not examine the effect of the RCT on participants who were depressed but who were not in therapy. Therefore, we do not know if the RCT would be effective for patients who could not secure a therapeutic relationship. Fourth, we do not know whether the treatment would be promising for patients who are not clinically depressed. Therefore, we cannot generalize the efficacy of the RCT to a sample of participants who are not clinically depressed and in therapy. Finally, as part of this RCT, we specifically designed materials for participants to reanalyze their hurtful interpersonal feelings and the support they received; these factors were designed as self-report diaries for participants in line with previous CBT for rumination. However, as we could not recruit enough participants to code these variables, we could not evaluate perceived hurtfulness and social support as potential mediators in the statistical analysis. Therefore, in subsequent work, there is a need to design better methods and measures of these potential mediators.

### Conclusions

This study is the first of its kind to focus on the episode-to-episode measurement, both at the individual client and group levels, of one specific outcome (depressive rumination) underlying depression. This pilot shows how promising this JITAI-MRFCBT approach is for identifying and blocking cascades of rumination in a timely way, providing appropriate CBT at the critical time of its need. “Seeing” the individual patient networks affords opportunities to better understand and mitigate rumination—a critical psychological process that exacerbates not only depression but also a range of mental health disorders and adverse health outcomes. Indeed, as others have noted, rumination appears to be a transdiagnostic process that provides insight across a large swath of disorders, making it particularly important to use tools such as those developed in this study to better understand these processes and mitigate their adverse effects—as doing so would appear to have broad generalizability. It is worth pointing out that, in our study, none of the users reported experiencing technical difficulties using their smartphones to access, navigate, and interact with the RCT website. Therefore, it speaks to the feasibility and scalability of delivering JITAI-MRFCBT without technical difficulties. As the initial pilot intervention was designed to reduce the complexity of installing apps and complicated interactive features, users were able to adhere to the intervention with minimal technical support. Most importantly, this design minimizes the requirements for specific types of smartphones. That is, a patient can have access to the RCT if their phone has access to the internet and a browser. As such, it is a promising approach in an era of increasingly personalized interventions that provide feedback and scaffolding for the client and their therapist and for researchers trying to better understand and mitigate adverse patterns of cognition, affect, and behavior across contexts and over time to advance wellness and this emerging field of rumination science.

## Abbreviations

CBT: cognitive behavioral therapy
CFI: comparative fit index
EMA: ecological momentary assessment
GIMME: Group Iterative Multiple Model Estimation
IRB: institutional review board
JITAI: just-in-time adaptive intervention
MCBT: mobile cognitive behavioral therapy
MDD: major depressive disorder
MRFCBT: mobile rumination-focused cognitive behavioral therapy
RCT: randomized controlled trial
REDCap: Research Electronic Data Capture
RFCBT: rumination-focused cognitive behavioral therapy
RMSEA: root mean square error of approximation
SDS: State Depression Scale
SRMR: standardized root mean square residual
uSEM: unified structural equation modeling
